# The role of personal factors in quality of life among Iranian women with vaginismus: a path analysis

**DOI:** 10.1186/s12955-021-01799-5

**Published:** 2021-06-15

**Authors:** Atefeh Velayati, Shahideh Jahanian Sadatmahalleh, Saeideh Ziaei, Anoshirvan Kazemnejad

**Affiliations:** 1 Department of Reproductive Health and Midwifery, Faculty of Medical Sciences, Tarbiat Modares University, Tehran, Iran; 2 Department of Biostatistics, Faculty of Medical Sciences, Tarbiat Modares University, Ale-Ahmad Highway, 14115-111 Tehran, Iran

**Keywords:** Vaginismus, Health-related quality of life, Anxiety, Depression, Path analysis

## Abstract

**Background:**

The aim of this study was to provide a path model for assessing the direct and/or indirect effects of psychological/behavioral parameters on health-related quality of life among women with vaginismus.

**Methods:**

A cross-sectional study was conducted on a sample of 236 women with vaginismus disorder attending to sex clinics in Tehran, Iran from April 2017 to March 2018. Data were collected using a demographic questionnaire, the marital satisfaction scale, the hospital anxiety and depression scale, the rosenberg self-esteem scale, the body image concern inventory, the short-form health survey (SF-12) and the female sexual quality of life questionnaire. In addition to descriptive statistical data, the fitness of the proposed model was investigated using path analysis.

**Results:**

The results of path analysis demonstrated that the final model had a good fit to the data (Chi-Square/degrees of freedom (Normed Chi^2^) = 2.12, root mean square error of approximation = 0.069, goodness fit index = 0.99, both comparative fit index = 0.99 and Tucker–Lewis index = 0.96). In this model, anxiety and depression significantly predicted health-related quality of life as measured by the SF-12.

**Conclusions:**

Anxiety and depression are important components in predicting health-related quality of life among those suffering from vaginismus.

**Supplementary Information:**

The online version contains supplementary material available at 10.1186/s12955-021-01799-5.

## Background

Vaginismus is defined as persistent or recurrent difficulties related to vaginal entry by penis, finger, and or any object, despite woman's desire [1]. Although the prevalence of the disorder is unknown, studies demonstrated that vaginismus is one of the most common sexual disorders among women [2, 3] that generally can influence health-related quality of life (HRQoL), well-being and sexual health [4, 5]. In the *Diagnostic and Statistical Manual of Mental Disorders Fifth Edition (DSM-5)*, vaginismus associated with dyspareunia has been defined as a single disorder called as 'genital-pelvic pain disorder' [1]. This disorder is culturally dependent, so its prevalence varies in different countries. For instance, vaginismus prevalence is estimated to be around 15.5% and 68% among studied samples in the United Kingdom and Ghana respectively [4, 6, 7]. There are few studies about vaginismus among Iranian women with a prevalence of 20.1% [8].

The association between women's sexual disorders and psychological problems such as depression and anxiety have been confirmed in various studies [9–12]. Vaginismus has adverse effects on personality, self-esteem, marital relationships and emotional stability of women [13, 14] Moreover, it has adverse effects on their male partners, who may suffer from sexual dysfunctions such as premature ejaculation and erectile dysfunction [15, 16]. A systematic review on vaginismus found that partners of women with vaginismus can be characterized as inactive and indecisive men who often suffer from sexual dysfunctions [2]. Many women with vaginismus have also shown low self-esteem, frustration, guilt [17, 18] and negative images of their body [18, 19]. Some women even have gone through divorce and have shown suicidal tendencies [20].

Women's sexual dysfunction has a major impact on HRQoL, sexual health and well-being [18, 21, 22]. Health-related quality of life is the health aspect of quality of life that focuses on people’s level of ability, daily functioning and ability to experience a fulfilling life [23]. In recent decades, in Iran, due to the increasing attention to the overall well-being, HRQoL is being considered as one of the most important factors in people's performance and efficiency [24]. Sexual quality of life (SQOL), on the other hand, is mainly related to the field of sexual and reproductive health and perception of the sexual aspect of life [23]. There are few studies assessing direct relationship between SQOL and psychological factors/behavioral parameters with ambiguous results [25]. However, when SQOL as a mediatory factor, or taking into account the effects of the existing independent variables in the network on each other, it becomes more difficult to examine the relationship between psychological/behavioral parameters and HRQoL. In fact, this framework indicates that psychological/behavioral parameters affect HRQoL directly and indirectly. This is mainly due to unmeasured inter-relationships and high collinearity, direct and indirect mechanisms, which exist between psychological factors/behavioral parameters and HRQoL. As a result, the association between psychological factors/behavioral parameters and HRQoL has not been well understood. Therefore, our study, of complex pathways by introducing the interrelated factor of SQOL, instead of assessing only the direct relationship between psychological factors/behavioral parameters and HRQoL, could help a better understanding of the role of intermediary variables in the evaluation of overall health outcomes.

Path analysis is a relatively novel technique in analyzing conceptual models by quantifying the relationships and interactions among a network of factors. The advantage of path analysis is the simultaneous assessment of all related pathways considering the role of independent and/or dependent, mediator factors in outcome development [26]. This approach utilizes multiple regression analysis simultaneously within the same analytical framework and models interactions between variables [27]. To the best of our knowledge, no previous study has evaluated direct and indirect associations between changeable risk factors of the SQOL on HRQoL simultaneously. Moreover, although interest in the health-related quality of life among women with sexual disorder(s) has recently increased, it has been largely ignored in Iranian research and literature. This is mainly because of cultural taboos around sexuality matters. Therefore, considering the importance of the issue and the little information about this field in Iran, it is necessary to identify the factors that directly and/or indirectly affect the health-related quality of life of women with vaginismus. In Iran, women are usually under intense pressure to get married. Having children is seen as the important indicator of a successful marriage. Premarital and extramarital sex is strictly forbidden because of religious sanctions and virginity is a vital cultural norm. It is expected that the first sexual intercourse, particularly for women, to take place after marriage and most often on the wedding night. Therefore, a wedding ceremony usually includes a white handkerchief and stains of blood are proof of bride's virginity on her wedding night. Lack of premarital experiences and information about sex causes significant stress and anxiety in newlywed women. This is far worse in newlywed with vaginismus, as the failure of having sexual encounters is often seen as woman's impotence and her lack of collaboration [28, 29].

Therefore, the present study designed a model with a multi-disciplinary approach for assessing the direct and/or indirect effects of psychological/behavioral parameters on health-related quality of life among women with vaginismus.

For designing this model, based on the background of research and review of studies, we used a set of variables such as anxiety, depression, self-esteem, marital satisfaction, poor body image and sexual quality of life simultaneously. After completing questionnaires related to each variable, the proposed model was evaluated using Statistical Package for Social Science (SPSS) 24.0 and path analysis with the AMOS 24.0. The results of the path analysis determined the exact effect of the predictor variables as well as the importance of each predictor variable in the model.

## Methods

### Theoretical framework

**Fig. 1:**
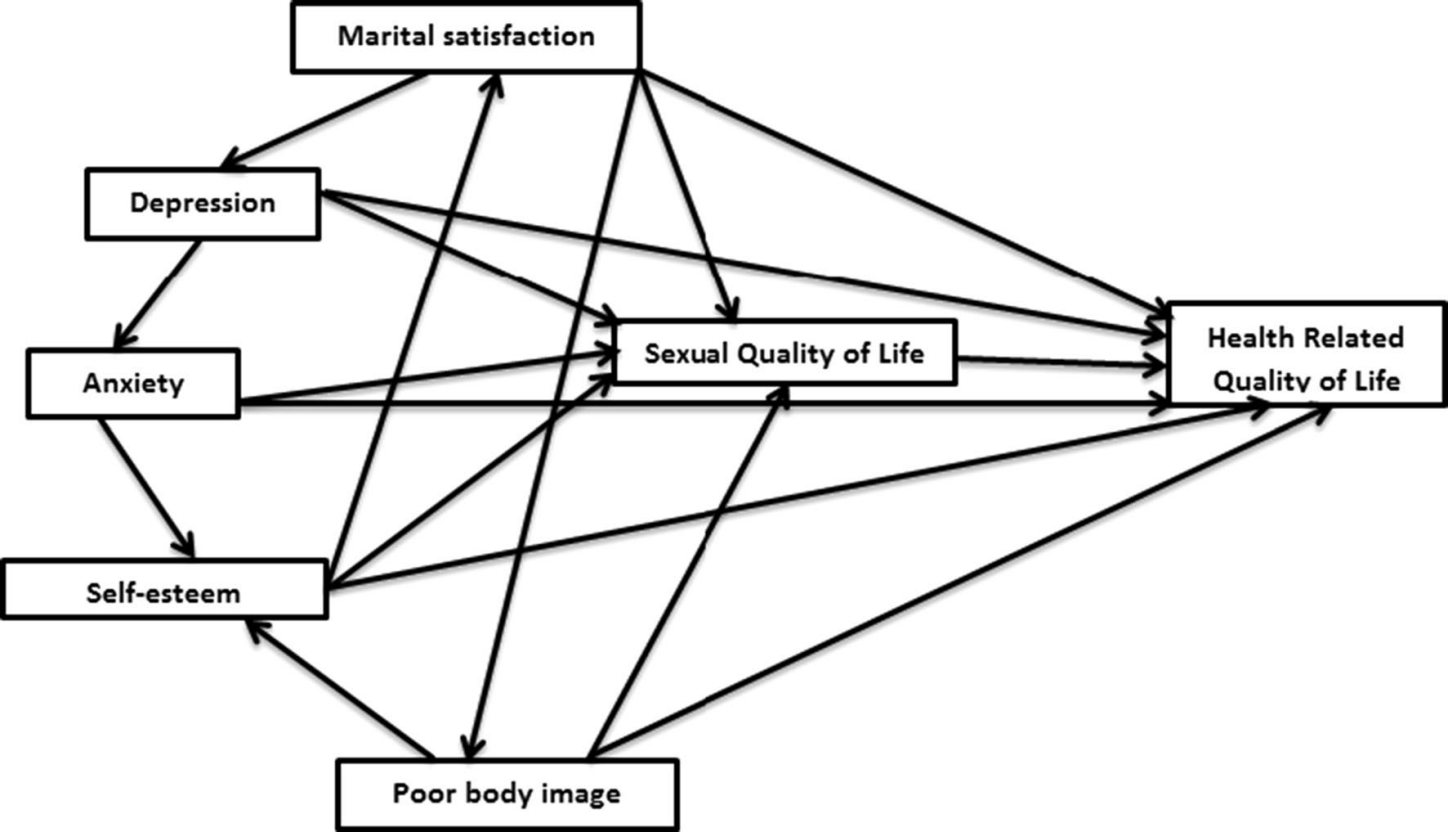
A path diagram. The straight arrows demonstrate regression paths for supposed causal relationships. The conceptual model indicates that psychological/behavioral parameters affect HRQoL directly and indirectly. Indirect effects occur when the relationship between two variables (e.g., anxiety and quality of Life) is mediated by one or more variables (i.e., sexual quality of Life and self-esteem)

At the first step, a conceptual model (Fig. 1) was developed based on information obtained from the literature [30–33]. After collecting the information from each variable's questionnaire, the proposed model was evaluated by using the AMOS software and path analysis method.

From April 2017 to March 2018, 256 women with vaginismus disorder were referred by a general practitioner to sex clinics located in Tehran, Iran. Despite the fact that some of the participants were able to conceive using infertility treatment or due to ejaculation near the outer part of the vagina, they all had not vaginal intercourse, for which they were seeking treatment. Their vaginismus diagnosis was initially identified through vaginal examination by experienced gynecologists. After assuring that they had no structural or physical complications, they were examined by psychologists to confirm their vaginismus disorder based on sexual history and indicators of Diagnostic and Statistical Manual of Mental Disorders—fourth Edition (DSM-IV). Having access to women with vaginismus disorder was facilitated through the clinical staff and specialists who explained the participants the purpose of the research and the importance of their contribution to the study through completing six sets of questionnaires. The participants of the study were selected through non-probability sampling.

In this cross-sectional study, after understanding the aim of the study, 240 women out of 256 women, agreed to join the study. The informed consent forms were obtained from all participants who gave permission for recording their demographic information. From 240 participants who signed the informed consent form, four participants refused to complete the questionnaires and were excluded from the study. Therefore, 236 women were included in this study. The inclusion criteria were (a) to be married for at least 6 months and be expected to be sexually active; (b) to be healthy, with no major mental or medical conditions (c) to be literate to understand and complete all the questionnaires independently and (d) not receiving any psychiatric or pharmacological treatments in the last 3 months.

### Study variables and measurements

#### Baseline information

Participants completed the following information at the start of the study: “age of the participant and her partner”, “education level of the participant and her partner (Less than Diploma, diploma, and university degree)”, “employment status of the participant and her partner (having any stipend job)”, “housing situation of the couple (rental, ownership)”, “duration of the disorder”, “having children (yes/ no)”, mode of birth (normal vaginal delivery, instrumental delivery and cesarean section)”, and their source of sexual information (friends, books, internet)”.

The following six sets of questionnaires were used to gather information related to the aim of the study.

##### Health-related quality of life (SF-12)

The Short-Form Health Survey (SF-12) [34], a shortened form of the SF-36 Health Survey (SF-36) [35], is a largely used instrument for assessing patient-reported general health conditions/HRQoL. The validity and reliability of the SF-12 have been documented in many studies (9, 13–15). The instrument is categorized in eight health domains to evaluate physical and mental health, each including six items. Physical health scales include general health (1 item), physical functioning (2 items), role physical (2 items), and bodily pain (1 item). Mental health domains include vitality (1 item), social functioning (1 item), role emotional (2 items) and mental health (2 items). Scores for items range from 1 to 6. To enable comparison of the study results in different cultures, we used US population-derived SF-12 norms which consider a mean value of 50 and a standard deviation value of 10 [36]. Scores on this questionnaire are in the range of 0–100, where higher scores indicate a better self-perceived health status. The validity and reliability of this questionnaire in Iran have been evaluated by Montazeri et al. [37].

##### Sexual quality of life questionnaire (SQOL-F)

The 18-item sexual quality of life-female (SQOL-F) questionnaire has been used to evaluate sexual health and wellbeing condition from women’s perspectives. Each item is rated with a 6 point Likert-like response scale (6 = “totally agree” to 1 = “totally disagree”). Scores on the SQOL-F range from 18 to 108 and higher scores of the questionnaire indicate a healthier sexual quality of life. The questionnaire has been translated from English to Farsi by Masoumi et al. and its psychometric properties evaluated in their study [38].

##### Marital satisfaction scale-shortened version (MSS)

This questionnaire contains 10 items measuring the satisfaction of marital relationships. Using the 5-point Likert scale, the answers range from “5 = I totally agree with” to “1 = I totally disagree with”. Total scoring of this questionnaire ranges from 10 to 50. The high scores indicate higher marital satisfaction [39]. A valid and reliable version of the MSS scale translated into Persian is available in Alidousti. et al. study [40].

##### Hospital anxiety and depression scale (HADS)

HADS scale is commonly used to evaluate levels of anxiety and depression. This 14-item self-assessment scale consists of seven questions for anxiety and seven questions for depression. HADS uses a four-point ordinal response format, ranges from 0 (non-appearance) to 3 (extreme appearance). We used standard scoring of the algorithms as HADS subscales. Anxiety score is a sum of responses for each of odd items (1^re^, 3^re^, 5^re^, 7, 9, 11^re^, and 13^re^) and depression scale is a sum of responses for each of even items (2, 4, 6^re^, 8 ^re^, 10^re^, 12, and 14), where superscripted “re” shows reverse-scored items. Scores on the subscales of hospital anxiety scale (HADS-a) and hospital depression scale (HADS-d) of this questionnaire are in the range of 0–21. Higher scores indicate a greater level of anxiety or depression. The psychometric properties of the Persian version of the questionnaire in Iran were assessed by Kaviani et al*.* [41].

##### Rosenberg self-esteem scale (RSES)

The SES questionnaire consists of 10 general questions that measure the degree of personal life satisfaction and good feelings. Responses for each item are scaled via a 4-point Likert scale ranging from 0 to 3. The scores range between a minimum value of 0 and a maximum value of 30, where higher score indicates better self-esteem. Validity and reliability of the Persian version of this questionnaire were evaluated by Kavyani et al. [42].

##### Body image concern inventory (BICI)

Body Image Concern Inventory is a commonly used questionnaire in health studies. This questionnaire contains 19 self-reported questions that assess appearance concerns. A 5-point liker scale ranging from 1 (never) to 5 (always) was used to assess the level of agreement with each question. The maximum score is 95 and the minimum score is 19, where higher scores signify a more negative mental image of the body. In Iran, the validity of this test was carried out by Besak Nejad and Ghaffari [43].

### Statistical analysis

IBM SPSS Amos version 24 was used to examine the fitting of HRQoL models to data obtained from the participants of this study. The data was described using mean (SD) for continuous variables and frequency (percentages) for categorical variables, respectively. To assess the links between marital satisfaction (MA), anxiety, depression, self-confidence, body image (BI), and sexual quality of life (SQOL) among women with Health-related quality of life (HRQoL), path analysis was used that enables researchers to test various regression equations. Following the completion of all sets of questionnaires, the conceptual model was presented using examples from a case study of path analysis to assess and estimate the direct, indirect, and total effects of predictors on HRQoL.

The independent variables for this study were: marital satisfaction, anxiety, depression, self-confidence, body image; and the dependent variable is the HRQoL measured by the SF-12. The SQOL had an intermediary effect in the association between psychological factors/behavioral parameters and HRQoL. After reviewing the theoretical foundations and formulating the selected theoretical framework, we first, designed the conceptual model of the research and then the fitting of the model paths was evaluated.

Model fit measures were obtained to conclude how well the suggested model caught the covariance among all the measures. The practical indicators of model fit are as follow: Chi-Square (χ^2^; p > 0.05), Chi-square/degrees of freedom (Normed χ^2^: CHI2/DF < 5), the root mean square error of approximation (RMSEA ≤ 0.08), the Comparative Fit Index (CFI ≥ 0.90), (GFI ≥ 0.90), Tucker–Lewis index (TLI ≥ 0.90) [44]. Regression coefficients were estimated using the Maximum Likelihood Estimation Procedure. *p* Values < 0.05 were considered statistically significant.

## Results

The mean (standard deviation) age of women with vaginismus disorder and their partners was 27.88 (5.72) and 31.48 (5.48) years old respectively. Most of participants (209 cases; 88.6%) had no children, apart from 27 women (11.4%). Most of the couples (154 cases: 65.25%) were not homeowners.

The highest self-reported duration of disorder was between 2 and 5 years (69 cases: 29.2%). The main source of sexual information for most of participants was the internet (164 cases: 72.2%); and most of participants (155 cases: 65.7%) were unemployed. The demographic information of participants is depicted in Table 1.

**Table 1:** Demographic information of couples and Midwifery profile of women with vaginismus disorder (n = 236)

Demographic information	Women, n (%)	Partners, n (%)
Age, mean (SD) (in years)	27.88 (5.72)	31.48 (5.48)
Less than diploma		
Diploma	12 (5.1)	28 (19.0)
University degree	13 (5.5)	15 (6.3)
University degree	211(89.4)	193 (81.7)
Employment status		
Unemployed	155 (65.7)	2 (0.8)
Employed	81 (34.3)	234 (99.2)
Housing situation		
Rental	154 (65.25)	
Ownership	82 (37.75)	
Duration of condition (in years)		
< 1	59 (25.0)	
1–2	65 (27.5)	
2–5	69 (29.2)	
5–10	33 (14.0)	
> 10	10 (4.2)	
Having a child		
Yes	27 (11.4)*	
No	209 (88.6)	
Women’s source of sexual information		
Friends	18 (7.6)	
Books	11 (4.7)	
Internet	164 (72.2)	
Friends, books, internet	19 (8.1)	
Friends, books	20 (8.5)	

All women with children had a c-section

The distribution of variables is presented in Table 2. The mean (SD) total HRQoL, SQOL, marital satisfaction and self-esteem scores of the participants were 43.7 (7), 57 (20), 31.5 (7.1) and 17.4 (5.5) respectively. The mean (SD) total anxiety, depression, and body image scores of them were 11.0 (4.5), 7.4 (4.3) and 41.5 (13.1) respectively (We presented the distribution of responses for all participants in the study for the instruments. Please see Additional file 1). The results show that almost scores of all variables are below the midpoint range.

**Table 2:** The status of the HRQoL, SQOL, marital satisfaction, depression, anxiety, self-esteem and body image concern (n = 236)

Indices (instrument)	Mean	Standard deviation	Minimum	Maximum
HRQOL (SF-12)	43.7	7	29	60
MCS	36.6	11.2	15	72
PCS	50.8	8.1	17	68
SQOL (SQOL-F)	57	20	18	108
Marital satisfaction (MSS)	31.5	7.1	13	45
Depression (HADS-d)	7.4	4.3	0	21
Anxiety (HADS-a)	11	4.5	0	21
Self-esteem (RSES)	17.4	5.5	2	29
Body image (BICI)	41.5	13.1	19	92

*PCS* physical component summary, *MCS* mental component summary

HRQoL ranges over (0–100) and SQOL ranges over (18–108); Marital satisfaction ranges over (10–50); depression ranges over (0–21); anxiety ranges over (0–21); self-esteem ranges over (0–30); BICI ranges over (19–95)

Table 3 presents the fit indices of predicting HRQoL model: (Normed Chi2 = 2.12 < 5.0, RMSEA = 0.069 < 0.080, GFI = 0.99, both CFI = 0.99 and TLI = 0.96 > 0.90).

**Table 3:** The goodness of fit indices for the models

	CFI	GFI	RMSEA	Chi-square	df	Chi-square/df	TLI
Path n = 236	0.99	0/99	0.069	4.23	2	2.12	0.96

*CFI* comparative fit index, *GFI* goodness fit index, *RMSEA* root mean square error of approximation, *Chi-square/df* chi-square to the degree of freedom index, *TLI* Tucker–Lewis index

Table 4 presents a summary of the path analysis standardized effects indicating the direct, indirect and total effects of the variables on the women’s HRQoL. In this model, depression and anxiety had directly significant and negative effect on participant’s HRQoL (*p* < 0.001). Findings also revealed that marital satisfaction (*p* = 0.002), depression (*p* = 0.017) and self-esteem (*p* < 0.001) had significantly indirect impact on women’s HRQoL. Estimated path coefficients indicated that HRQoL was largely predicted by depression (β = − 0.387) and anxiety (β = − 0.227) and was not influenced by Body image and SQOL. Whereas HRQoL was directly predicted by depression and anxiety, marital satisfaction (β = 0.276) and self-esteem (β = 0.143) had an indirect effect on HRQoL. In addition to the direct effect of depression, it also indirectly affected HRQoL. Finally as the table indicated, while the relationship between HRQoL with marital satisfaction and self-esteem were positive it was negatively associated with depression and anxiety.

**Table 4:** Standardized effects of study variables on the health-related quality of life in a woman with vaginismus disorder (n = 236)

Variable	Direct effects	Indirect effects	Total effects
Marital satisfaction	0.064 (− 0.374 to 0.070)	0.276* (0.266 to 0.286)	0.340 (− 0.06 to 0.74)
Depression	− 0.387* (− 0.997 to − 0.375)	− 0.200* (− 0.204 to − 0.196)	− 0.587* (− 0.610 to − 0.564)
Anxiety	− 0.227* (− 0.638 to − 0.273)	− 0.068 (− 0.075 to − 0.061)	− 0.296* (− 0.307 to − 0.285)
Self-esteem	0.085 (− 0.372 to 0.092)	0.143* (0.140 to 0.146)	0.229 (− 0.171 to 0.629)
Body image	− 0.035 (− 0.135 to 0.065)	− 0.057 (− 0.121 to 0.007)	− 0.092 (− 0.242 to 0.058)
SQOL	0.089 (− 1.365 to 0.159)	–	0.089 (− 0.112 to 0.289)

All values are presented as estimates of standardized effects (95% CI)

^*^Significant (*p* < 0.05)

**Fig. 2:**
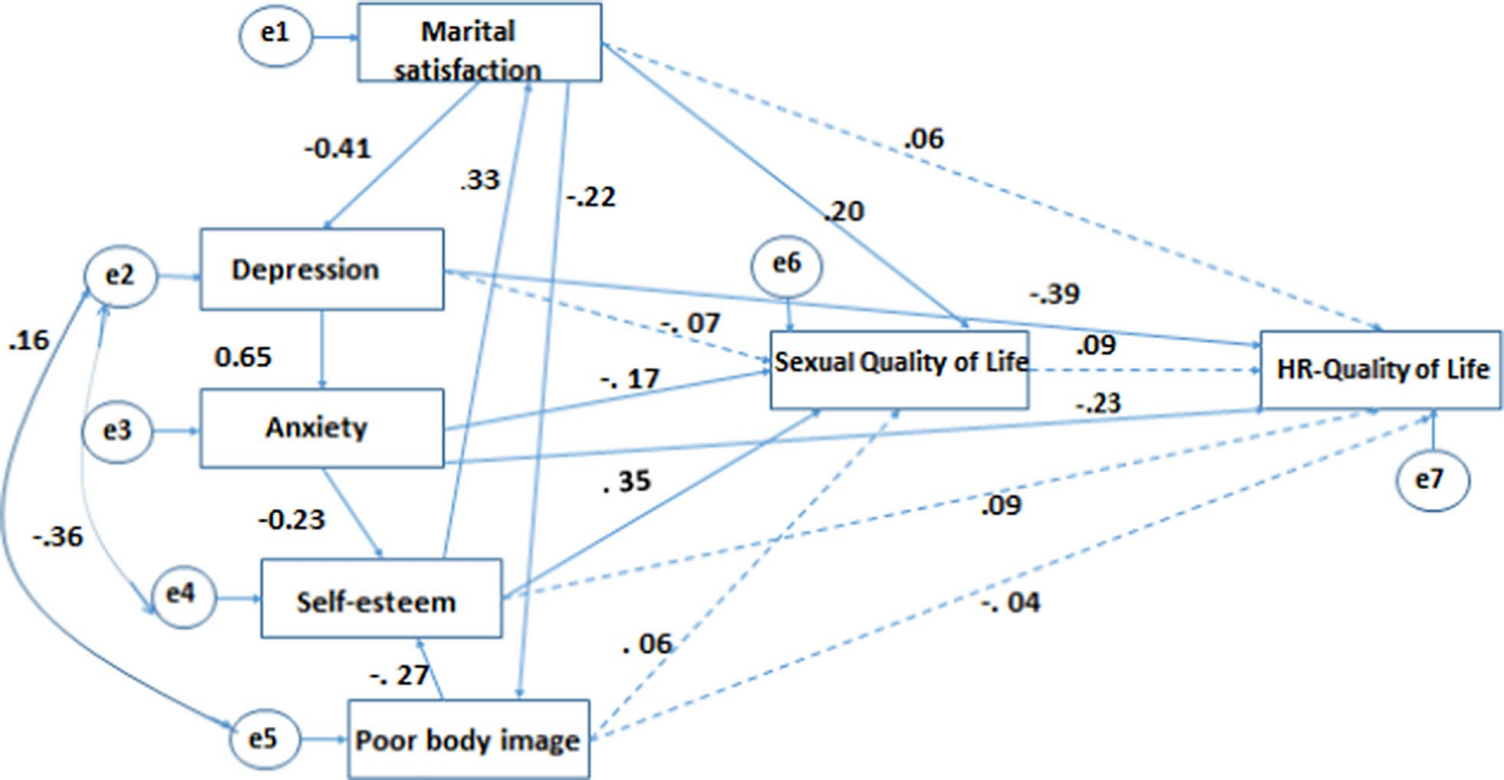
Path diagram of the relationship of Health-related quality of life and sex predictors. Values represent standardized regression coefficients. Arrows between two variables (pathways) indicated that they are correlated and the direction of correlation. The e1 through e7 stands for error measurement (unreliability) in each item. All path with solid lines were statistically significant (All *p* < 0.05)

Figure 2 presents the conceptual model which was examined by path analysis. The data supports the conceptual model with covariance between depression and body image concern as well as depression and self-esteem. As it has been indicated in the Fig. 2, independent variables not only had effect on HRQoL, but also affected each other. In summary, the findings demonstrated that psychological/behavioral parameters significantly had direct and/or indirect effects on HRQoL.

## Discussion

This study aimed to evaluate the direct and/or indirect effects of psychological/behavioral parameters on Health-related quality of life among women with vaginismus.

The selection of the main predictors of HRQoL (anxiety, depression, self-esteem, and body image concern, marital satisfaction and SQOL) was based on findings from previous studies [45–49]. To assess the conceptual model, a path analysis was conducted. The results showed that the final model had a good fit on data. The finding indicated that HRQoL was largely predicted by depression and anxiety, marital satisfaction and self-esteem and was not influenced by Body image and SQOL. In addition independent variables not only had positive or negative, direct and/or indirect, impact on HRQoL, but also affected each other.

The relationship between marital satisfaction and HRQoL was mediated by depression and SQOL. Moreover, self-esteem and HRQoL were mediated by marital satisfaction, depression level and SOQL. All of the above results indicate that depression directly or as a mediating variable has a key role in the HRQoL of women with vaginismus.

The finding showed a high level of anxiety was important factor to predict poor HRQoL directly. It also mediated the relationship between marital satisfaction as well as depression with HRQoL. Scientific literature also confirms the important role of anxiety and depression in predicting HRQoL [50, 51]. In studies by Akyol et al. [52], Sadoghi and Mohammad Salehi [53], and Sarma and Byrne [54] a significant relationship anxiety and depression with HRQoL was confirmed. In Kim and Kang's study, depression was a predictor of HRQoL [55]. Anxiety and depression reduce HRQoL through various potential mechanisms. Physiologically, anxiety and depression motivate the sympathetic nervous system, increase the heart rate, impair platelet function, stimulate the inflammatory process, and cause hypercholesterolemia [56, 57]. Behaviorally, anxious and depressed patients disregard their care and diet and do not follow the prescribed medication [58].

Women with sexual dysfunction develop symptoms of mood instability, low self-esteem, anxiety, selfishness and guilt [59]. They do not express their feelings and desires [60] and lose their attachment to life, work, and other activities that were enjoyable for them previously [61]. Barlow believes that factors such as the expectation of failure, concerns about negative sexual partner evaluation, and concerns about sexual response may cause anxiety that can lead to further failure and prevent sexual intercourse [62].

Given the association between anxiety and depression with vaginismus [63–65], the devastating effects of vaginismus on women [14, 66, 67], the prevalence of dissatisfaction with sexual life among women with vaginismus, and the view that SQOL an important dimension of women's HRQoL [68], it can be concluded that anxiety and depression caused by vaginismus disorder decrease the HRQoL among this group of women.

In our findings, the scores of SQOL and marital satisfaction were just above average. This may be because of the duration of disorder was less than 2 years for more than half of participants of the study. However, as the problem persists, their SQOL and marital satisfaction are expected to decline considerably. Although, the average SQOL score of this study was slightly higher than the midpoint potential range, it was lower than the SQOL score reported for healthy women in other studies. Indeed result of studies show a relation between sexual disorders with poor SQOL, which ultimately affects the health-related quality of life, adversely; confirming our findings [69, 70].

The positive and negative effects of a person's physical image also affect their HRQoL and self-esteem [71]. Sexual attitudes are also associated with one's body image so that people with good body image can easily accept their sexual expectations [72]. Therefore, if a person's body image is highly inconsistent, the self-esteem is often reduced [73]. As a result, their social relationships, daily functioning, interpersonal relationships, family relationships, and ultimately marital relationships are among the domains affecting their HRQoL. For instance, negative thoughts about body image are significantly correlated with sexual dissatisfaction. Findings from studies by Nobre and Pinto-Gouveia have shown that women with sexual dysfunction present more negative sexual beliefs, especially negative body images [74, 75], however in our study, body image did not associate with SQOL.

Marital satisfaction is one of the vital factors affecting family stability and has an important role in health, reducing the psychological problems of couples and increasing their life expectancy. Similarly, dissatisfaction with marital life is associated with several devastating consequences, such as depression and self-esteem in couples. Sexual satisfaction, as vital aspect of marital satisfaction also affects the quality of couples' relationships. Experience of sexual satisfaction increases intimacy in the relationship and reduces relationship stress [76]. In our study, there are indirectly positive effects of marital satisfaction and self-esteem on HRQoL. Similar results were also obtained by BagarozziL [76] yasand et al. [77] and Al-Darmaki et al. [78].

Infertility is a major concern for women suffering from vaginismus because of not being able to have sexual intercourse [79–81]. In our study, there were cases of women with children that indicate pregnancy is still possible among women with vaginismus and that they could benefit from fertility treatments.

## Strengths and limitations of the study

This study has several strengths. We recruited a large population of women with vaginismus. We designed a model with a multi-disciplinary approach for assessing the effects of psychological/behavioral parameters on health-related quality of life among women with vaginismus. We found not only the direct effects of predictor variables on the HRQoL, but also we found their indirect and total effects.

One of the limitations of this study is that only women diagnosed with vaginismus who were referred to sex treatment clinics were included in this study. In other words, women who did not seek treatment were not examined. In this study, demographic factors had not been included in the conceptual model to understand the association of the demographic factors and health-related quality of life or sexual quality of life which should be explored in further studies. This study was also conducted among urban women only. Therefore, the results are not generalizable to all women with this disorder. For this reason, it is recommended to also carry out this study among affected women in rural areas.

## Conclusions

Depression and anxiety were two important variables that affected the sexual functioning (SQOL) of participants of this study. Therefore, in the treatment of sexual dysfunction, it is also better to pay more attention to these variables. The finding of the present study is suggested to be used as a basis for designing appropriate and effective interventions to prevent psychological disorders, especially anxiety and depression among women with vaginismus disorder.

## Supplementary Information

**Additional file 1.** The distribution of responses for all participants in the study for the instruments.

## Data Availability

Data supporting our findings can be made available upon request.

## Abbreviations

HRQoL: health-related quality of life
SQOL: sexual quality of life
SF-12: 12-item short-form health survey
SQOL-F: sexual quality of life questionnaire for a female
MSS: marital satisfaction questionnaire-shortened version
HADS: hospital anxiety and depression scale
HADS-d: hospital anxiety and depression scale’s scales for depression
HADS-a: hospital anxiety and depression scale’s scales for anxiety
RSES: rosenberg self-esteem scale
BICI: body image concern inventory
χ^2^: chi-square
Normed χ^2^: chi-square/degrees of freedom
RMSEA: root mean square error of approximation
CFI: comparative fit index
GFI: goodness fit index
TLI: Tucker–Lewis index
