# Supplementary material for: The role of personal factors in quality of life among Iranian women with vaginismus: a path analysis
Source: Health Qual Life Outcomes. 2021 Jun 15;19:166. doi: 10.1186/s12955-021-01799-5 (PMC8204437; doi:10.1186/s12955-021-01799-5)
Supplement: Supplementary file 1 — Additional file 1. The distribution of responses for all participants in the study for the instruments. [file 12955_2021_1799_MOESM1_ESM.docx]

| **Body Image Concern Inventory** | | | | | | | | | | |
| --- | --- | --- | --- | --- | --- | --- | --- | --- | --- | --- |
| **Always** | | **Often** | | **Sometimes** | | **Rarely** | | **Never** | |  |
| Percent | No | Percent | No | Percent | No | Percent | No | Percent | No |  |
| 6.4 | 15 | 15.7 | 37 | 28 | 66 | 30.1 | 71 | 19.9 | 47 | 1. I am dissatisfied with some aspect of my appearance |
| 7.2 | 17 | 12.3 | 29 | 27.1 | 64 | 38.1 | 90 | 15.3 | 36 | 2. I spend a significant amount of time checking my appearance in the mirror |
| 7.2 | 17 | 9.7 | 23 | 17.4 | 41 | 25.4 | 60 | 25.4 | 95 | 3. I feel others are speaking negatively of my appearance |
| 7.6 | 18 | 19.9 | 47 | 18.2 | 43 | 19.9 | 47 | 34.3 | 81 | 4. I am reluctant to engage in social activities when my appearance does not meet my satisfaction |
| 6.8 | 16 | 9.7 | 23 | 24.2 | 57 | 33.9 | 80 | 25.4 | 60 | 5. I feel there are certain aspects of my appearance that are extremely unattractive |
| 11.0 | 26 | 18.2 | 43 | 27.5 | 65 | 24.6 | 58 | 18.6 | 44 | 6. I buy cosmetic products to try to improve my appearance |
| 5.1 | 12 | 17.8 | 42 | 21.6 | 51 | 25.8 | 61 | 29.7 | 70 | 7. I seek reassurance from others about my appearance |
| 5.5 | 13 | 11.9 | 28 | 23.3 | 55 | 27.5 | 65 | 31.8 | 75 | 8. I feel there are certain aspects of my appearance I would like to change |
| 5.1 | 12 | 8.5 | 20 | 14.4 | 34 | 23.3 | 55 | 48.7 | 115 | 9. I am ashamed of some part of my body |
| Never: 1 Rarely:2 Sometimes:3 Often:4 Always:5  **Body Image Concern Inventory(continue)** | | | | | | | | | | |
| Percent | No | Percent | No | Percent | No | Percent | No | Percent | No |  |
| 3.4 | 8 | 12.7 | 30 | 22.0 | 52 | 29.2 | 69 | 32.6 | 77 | 10. I compare my appearance to that of fashion models or others |
| 7.2 | 17 | 12.7 | 30 | 25.8 | 61 | 33.5 | 79 | 20.8 | 49 | 11. I try to camouflage certain flaws in my appearance |
| 8.1 | 19 | 15.7 | 37 | 28.0 | 66 | 29.7 | 70 | 18.6 | 44 | 12 .I examine flaws in my appearance |
| 9.3 | 22 | 18.2 | 43 | 19.5 | 46 | 23.3 | 55 | 29.7 | 70 | 13. I have bought clothing to hide a certain aspect of my appearance |
| 5.5 | 13 | 12.3 | 29 | 29.2 | 69 | 33.1 | 78 | 19.9 | 47 | 14. I feel others are more physically attractive than me |
| 4.7 | 11 | 2.5 | 6 | 15.3 | 36 | 25.0 | 59 | 52.5 | 124 | 15. I have considered consulting/consulted some sort of medical expert regarding flaws in my appearance |
| 2.1 | 5 | 5.1 | 12 | 2.5 | 6 | 16.1 | 38 | 74.2 | 175 | 16. I have been embarrassed to leave the house because of my appearance |
| .4 | 1 | 1.3 | 3 | 3.8 | 9 | 6.8 | 16 | 87.7 | 207 | 17. I fear that others will discover my flaws in appearance |
| 3.0 | 7 | 5.1 | 12 | 6.8 | 16 | 22.0 | 52 | 63.1 | 149 | 18. I have missed social activities because of my appearance |
| .4 | 1 | 1.7 | 4 | 2.1 | 5 | 7.2 | 17 | 88.6 | 209 | 19. I have avoided looking at my appearance in the mirror |
| BICI ranges over (19- 95). | | | | | | | | | | |

| **Rosenberg's Self-Esteem Scale** | | | | | | | | |
| --- | --- | --- | --- | --- | --- | --- | --- | --- |
| **Strongly disagree** | | **Disagree** | | **Agree** | | **Strongly agree** | |  |
| Percent | No | Percent | No | Percent | No | Percent | No |  |
| 4.2 | 10 | 18.6 | 44 | 58.1 | 137 | 19.1 | 45 | 1. On the whole, I am satisfied with myself |
| 14.0 | 33 | 39.4 | 93 | 38.1 | 90 | 8.5 | 20 | 2. At times I think I am no good at all. |
| 1.3 | 3 | 4.2 | 10 | 48.7 | 115 | 45.8 | 108 | 3. I feel that I have a number of good qualities |
| .8 | 2 | 18.6 | 44 | 46.2 | 109 | 34.3 | 81 | 4.I am able to do things as well as most other people. |
| 17.8 | 42 | 48.3 | 114 | 25.8 | 61 | 8.1 | 19 | 5.I feel I do not have much to be proud of. |
| 9.7 | 23 | 29.2 | 69 | 45.3 | 107 | 15.7 | 37 | 6. I certainly feel useless at times. |
| 3.4 | 8 | 26.3 | 62 | 44.5 | 105 | 25.8 | 61 | 7. feel that I am a person of worth, at least on an equal plane with others. |
| 7.6 | 18 | 13.1 | 31 | 45.3 | 107 | 33.9 | 80 | 8. I wish I could have more respect for myself. |
| 16.9 | 40 | 38.1 | 90 | 31.8 | 75 | 13.1 | 31 | 9. All in all, I am inclined to feel that I am a failure. |
| 5.5 | 13 | 25.8 | 61 | 48.7 | 115 | 19.9 | 47 | 10. I take a positive attitude toward myself. |
| Self-Esteem ranges over (0- 30); Scores are calculated as follows: Strongly agree = 0 Agree = 1 Disagree = 2 Strongly disagree = 3  For items 1, 3, 4, 7, and 10, the coding was reversed. | | | | | | | | |

| **Sexual Quality of Life–Female Questionnaire** | | | | | | | | | | | | |
| --- | --- | --- | --- | --- | --- | --- | --- | --- | --- | --- | --- | --- |
| totally agree | | totally agree | | totally agree | | totally agree | | totally agree | | totally agree | |  |
| Percent | No | Percent | No | Percent | No | Percent | No | Percent | No | Percent | No |  |
| 5.9 | 14 | 11.0 | 26 | 12.3 | 29 | 20.8 | 49 | 20.8 | 49 | 29.2 | 69 | 1. When I think about my sexual life, it is an enjoyable part of my life overall. |
| 13.6 | 32 | 12.3 | 29 | 9.7 | 23 | 19.5 | 46 | 24.6 | 58 | 20.3 | 48 | 2. When I think about my sexual life, I feel frustrated. |
| 12.3 | 29 | 13.1 | 31 | 10.2 | 24 | 22.9 | 54 | 21.2 | 50 | 20.3 | 48 | 3. When I think about my sexual life, I feel depressed. |
| 8.9 | 21 | 8.9 | 21 | 9.7 | 23 | 23.3 | 55 | 21.6 | 51 | 27.5 | 65 | 4. When I think about my sexual life, I feel like less of a woman. |
| 16.5 | 39 | 24.6 | 58 | 27.5 | 65 | 13.6 | 32 | 10.6 | 25 | 7.2 | 17 | 5.When I think about my sexual life, I feel good about myself. |
| 8.5 | 20 | 7.6 | 18 | 11.4 | 27 | 27.5 | 65 | 18.6 | 44 | 26.3 | 62 | 6. I have lost confidence in myself as a sexual partner. |
| 7.2 | 17 | 10.2 | 24 | 5.1 | 12 | 26.3 | 62 | 25.4 | 60 | 25.8 | 61 | 7. When I think about my sexual life, I feel anxious. |
| 10.6 | 25 | 15.3 | 36 | 8.9 | 21 | 22.0 | 52 | 25.0 | 59 | 18.2 | 43 | 8. When I think about my sexual life, I feel angry. |
| 6.4 | 15 | 14.8 | 35 | 16.9 | 40 | 18.2 | 43 | 21.2 | 50 | 22.5 | 53 | 9. 9. When I think about my sexual life, I feel close to my partner. |

| **Sexual Quality of Life–Female Questionnaire(countinue)** | | | | | | | | | | | | |
| --- | --- | --- | --- | --- | --- | --- | --- | --- | --- | --- | --- | --- |
| Totally disagree | | disagree | | totally agree | | totally agree | | agree | | totally agree | |  |
| percent | no | percent | no | percent | no | percent | no | percent | no | percent | no |  |
| 5.1 | 12 | 5.9 | 14 | 8.5 | 20 | 16.5 | 39 | 23.3 | 55 | 40.7 | 96 | 10. I worry about the future of my sexual life. 3.0 2.8 4.6∗ |
| 9.3 | 22 | 11.4 | 27 | 11.9 | 28 | 20.3 | 48 | 24.6 | 58 | 22.5 | 53 | 11. I have lost pleasure in sexual activity. |
| 13.1 | 31 | 11.9 | 28 | 9.3 | 22 | 19.1 | 45 | 18.2 | 43 | 28.4 | 67 | 12. When I think about my sexual life, I am embarrassed. |
| 4.2 | 10 | 9.3 | 22 | 8.1 | 19 | 17.8 | 42 | 23.7 | 56 | 36.9 | 87 | 13. When I think about my sexual life, I feel that I can talk to my partner about sexual matters. |
| 17.4 | 41 | 16.1 | 38 | 12.3 | 29 | 21.6 | 51 | 17.8 | 42 | 14.8 | 35 | 14. I try to avoid sexual activity. |
| 23.3 | 55 | 14.0 | 33 | 10.2 | 24 | 11.9 | 28 | 18.6 | 44 | 22.0 | 52 | 15. When I think about my sexual life, I feel guilty. |
| 13.1 | 31 | 8.9 | 21 | 4.7 | 11 | 16.9 | 40 | 22.5 | 53 | 33.9 | 80 | 16. When I think about my sexual life, I worry that my partner feels hurt or rejected. |
| 14.0 | 33 | 9.3 | 22 | 5.5 | 13 | 14.0 | 33 | 24.6 | 58 | 32.6 | 77 | 17. When I think about my sexual life, I feel like I have lost something |
| 26.7 | 63 | 27.5 | 65 | 15.3 | 36 | 12.7 | 30 | 11.0 | 26 | 6.8 | 16 | 18. When I think about my sexual life, I am satisfied with the frequency of sexual activity. |
| Each item is rated with a 6 point Likert-like response scale (1 = "totally agree" to 6 = "totally disagree"). Scores on the SQOL-F range from 18 to108. For items 1, 5, 9, 13, and 18, the coding was reversed. | | | | | | | | | | | | |

| **Short Form Health Survey (SF-12)** | | | | | | | | | | | | |
| --- | --- | --- | --- | --- | --- | --- | --- | --- | --- | --- | --- | --- |
| poor | | fair | | good | | Very good | | | Excellent | | | 1. In general, you would say your health is? |
| Percent | No | Percent | No | Percent | No | Percent | No | | Percent | No | |  |
| 8 | 2 | 19.5 | 46 | 38.6 | 91 | 28.4 | 67 | | 12.7 | 30 | |  |
| The following questions are about activities you might do during a typical day. Does your health now limit you in  these activities? If so, how much | | | | | | | | | | | | |
| NO, not limited at all | | | | Yes, limited a little | | | Yes, a limited a lot | | | | 2. Moderate activities such as moving a  table, pushing a vacuum cleaner, bowling, or playing golf. | |
| Percent | | No | | Percent | No | | Percent | No | | |  |  |
| 76.2 | | 180 | | 17.8 | 42 | | 5.9 | 14 | | |  |  |
| NO, not limited at all | | | | Yes, limited a little | | | Yes, a limited a lot | | | | 3. Climbing several flights of stairs. | |
| Percent | | No | | Percent | No | | Percent | No | | |  |  |
| 75.4 | | 178 | | 19.5 | 46 | | 5.1 | 12 | | |  |  |
| During the past 4 weeks, have you had any of the following problems with your work or other regular daily activities as a result of your physical health? | | | | | | | | | | | | |
| never | | Seldom | | Some times | | Most times | | | All times | | | 4. Accomplished less than you would like. |
| Percent | No | Percent | No | Percent | No | Percent | No | | Percent | No | |  |
| 26.7 | 63 | 27.1 | 64 | 23.3 | 55 | 19.1 | 45 | | 3.8 | 9 | |  |
| never | | Seldom | | Sometimes | | Most times | | | All times | | | 5. Were limited in the kind of work or other activities. |
| Percent | No | Percent | No | Percent | No | Percent | No | | Percent | No | |  |
| 34.7 | 82 | 24.2 | 57 | 24.6 | 58 | 14.8 | 35 | | 1.7 | 4 | |  |
| During the past 4 weeks, have you had any of the following problems with your work or other regular daily activities as a result of any emotional problems (such as feeling depressed or anxious)? | | | | | | | | | | | | |
| never | | Seldom | | Some times | | Most times | | | All times | | | 6. Accomplished less than you would like |
| Percent | No | Percent | No | Percent | No | Percent | No | | Percent | No | |  |
| 12.7 | 30 | 24.2 | 57 | 32.2 | 76 | 24.6 | 58 | | 6.4 | 15 | |  |
| never | | Seldom | | Sometimes | | Most times | | | All times | | | 7. Did work or activities less carefully than usual. |
| Percent | No | Percent | No | Percent | No | Percent | No | | Percent | No | |  |
| 14.4 | 34 | 24.2 | 57 | 33.1 | 78 | 23.7 | 56 | | 4.7 | 11 | |  |
| Extremely | | Quite a bit | | moderately | | A little bit | | | Not at all | | | 8. During the past 4 weeks, how much did pain interfere with your normal work (including work outside the home and housework)? |
| Percent | No | Percent | No | Percent | No | Percent | No | | Percent | No | |  |
| 2.1 | 5 | 10.6 | 25 | 20.3 | 48 | 27.5 | 65 | | 39.4 | 93 | |  |
| These questions are about how you have been feeling during the past 4 weeks. For each question, please give the one answer that comes closest to the way you have been feeling. How much of the time during the past 4 weeks? | | | | | | | | | | | | |
| never | | Seldom | | Some times | | Most times | | | All times | | | 9. Have you felt calm & peaceful? |
| Percent | No | Percent | No | Percent | No | Percent | No | | Percent | No | |  |
| 2.5 | 6 | 17.8 | 42 | 27.5 | 65 | 30.6 | 84 | | 16.5 | 39 | |  |
| never | | Seldom | | Sometimes | | Most times | | | All times | | | 10. Did you have a lot of energy? |
| Percent | No | Percent | No | Percent | No | Percent | No | | Percent | No | |  |
| 7.6 | 18 | 30.5 | 72 | 30.9 | 73 | 25 | 59 | | 5.9 | 14 | |  |
| never | | Seldom | | Some times | | Most times | | | All times | | | 11. Have you felt down-hearted and blue? |
| Percent | No | Percent | No | Percent | No | Percent | No | | Percent | No | |  |
| 5.5 | 13 | 13.1 | 31 | 36 | 85 | 35.2 | 83 | | 10.2 | 24 | |  |
| never | | Seldom | | Some times | | Most times | | | All times | | | 12. During the past 4 weeks, how much of the time has your physical health or emotional has limited your social activity ( like visiting your friends, family , etc.) |
| Percent | No | Percent | No | Percent | No | Percent | No | | Percent | No | |  |
| 18.6 | 44 | 24.2 | 57 | 33.5 | 79 | 19.1 | 45 | | 4.7 | 11 | |  |
| Scores on this questionnaire are in the range of 0 to 100, where higher scores indicate a better self-perceived health status. | | | | | | | | | | | | |

| **Marital Satisfaction Scale-shortened version (MSS)** | | | | | | | | | | |
| --- | --- | --- | --- | --- | --- | --- | --- | --- | --- | --- |
| Strongly disagree | | Disagree | | neither agree nor disagree | | agree | | Strongly agree | |  |
| percent | no | percent | no | percent | no | percent | no | percent | no |  |
| 25 | 59 | 28.4 | 67 | 27.5 | 65 | 13.6 | 32 | 5.5 | 13 | 1-I am not pleased with the personality characteristics and personal habits of my partner. |
| 4.7 | 11 | 12.7 | 30 | 24.2 | 57 | 32.6 | 77 | 25.8 | 61 | 2- I am very happy with how we handle role responsibilities in our marriage. |
| 32.6 | 77 | 26.7 | 63 | 14.8 | 35 | 14.8 | 35 | 11 | 26 | 3- I am not happy about our communication and feel my partner does not understand me. |
| 6.4 | 15 | 15.7 | 37 | 25 | 59 | 30.9 | 73 | 22 | 52 | 4- I am very happy about how we make decisions and resolve conflicts. |
| 23.7 | 56 | 32.2 | 76 | 16.5 | 39 | 19.1 | 45 | 8.5 | 20 | 5- I am unhappy about our financial position and the way we make financial decisions. |
| 6.8 | 16 | 14.4 | 34 | 19.9 | 47 | 30.9 | 73 | 28 | 66 | 6- I am very happy with how we manage our leisure activities and the time we spend together. |
| 7.6 | 18 | 16.5 | 39 | 25 | 59 | 28.8 | 68 | 22 | 52 | 7- I am very pleased about how we express affection and relate sexually. |
| 0.8 | 2 | 3 | 7 | 2.5 | 6 | 0.8 | 2 | 2.5 | 6 | 8- I am not satisfied with the way we each handle our responsibilities as parents. |
| 15.7 | 37 | 18.2 | 43 | 24.6 | 58 | 25.8 | 61 | 15.7 | 37 | 9- I am dissatisfied about our relationship with my parents, in-laws, and/or friends. |
| 5.5 | 13 | 8.5 | 20 | 19.9 | 47 | 39.4 | 93 | 26.7 | 63 | 10- I feel very good about how we each practice our religious beliefs and values. |
| Marital satisfaction ranges over (10-50); 1-Strongly agree 2- agree 3-Neither Agree nor Disagree 4- Disagree 5-Strongly Disagree  For items 2, 4, 6,7, and 10, the coding was reversed. | | | | | | | | | | |

| **Hospital Anxiety and Depression Scale (HADS)** | | | | | | | | |
| --- | --- | --- | --- | --- | --- | --- | --- | --- |
| Not at all | | From time to time ,occasionally | | A lot of the time | | Most of the time | | 1. I feel tense or 'wound up': |
| Percent | No | Percent | No | Percent | No | Percent | No |  |
| 11.4 | 27 | 46.6 | 11o | 21.2 | 50 | 20.8 | 49 |  |
| Hardly at all | | Only a little | | Not quite so much | | Definitely as much | | 2. I still enjoy the things I used to enjoy: |
| Percent | No | Percent | No | percent | No | Percent | No |  |
| 3.8 | 9 | 14.00 | 33 | 59.3 | 140 | 22.9 | 54 |  |
| Not at all | | A little, but it doesn't worry me | | Yes, but not too badly | | Very definitely and quite badly | | 3. I get a sort of frightened feeling as if something awful is about to happen: |
| Percent | No | Percent | No | Percent | No | Percent | No |  |
| 12.3 | 29 | 12.7 | 30 | 48.3 | 114 | 26.7 | 63 |  |
| Not at all | | Definitely not so much now | | Not quite so much now | | As much as I always could | | 4. I can laugh and see the funny side of things: |
| Percent | No | Percent | No | Percent | No | Percent | No |  |
| 3.00 | 7 | 20.3 | 48 | 53.4 | 126 | 23.3 | 55 |  |
| Only occasionally | | From time to time , but not too often | | A lot of the time | | A great deal of the time | | 5. Worrying thoughts go through my mind: |
| Percent | No | Percent | No | Percent | No | Percent | No |  |
| 10.2 | 24 | 31.8 | 75 | 37.7 | 89 | 20.3 | 48 |  |
| Most of the time | | Sometimes | | Not often | | Not at all | | 6. I feel cheerful |
| Percent | No | Percent | No | Percent | No | Percent | No |  |
| 22.5 | 53 | 42.4 | 100 | 28.8 | 68 | 6.4 | 15 |  |
| Not Often | | Not at all | | Usually | | Definitely | | 7. I can sit at ease and feel relaxed: |
| Percent | No | Percent | No | Percent | No | Percent | No |  |
| 5.9 | 14 | 32.2 | 76 | 49.6 | 117 | 12.3 | 29 |  |
| Not at all | | Sometimes | | Very often | | Nearly all the time | | 8. I feel as if I am slowed down: |
| Percent | No | Percent | No | Percent | No | Percent | No |  |
| 27.1 | 64 | 40.7 | 96 | 20.8 | 49 | 11.4 | 27 |  |
| Very Often | | Quite Often | | Occasionally | | Not at all | | 9.I get a sort of frightened feeling like 'butterflies' in the stomach: |
| Percent | No | Percent | No | Percent | No | Percent | No |  |
| 25.4 | 60 | 25 | 59 | 43.2 | 102 | 6.4 | 15 |  |

| **Hospital Anxiety and Depression Scale (HADS) (continue)** | | | | | | | | |
| --- | --- | --- | --- | --- | --- | --- | --- | --- |
| I take just as much care as ever | | I may not take quite as much care | | I don't take as much care as I should | | Definitely | | 10. I have lost interest in my appearance: |
| Percent | No | Percent | No | percent | no | percent | no |  |
| 31.8 | 75 | 27.5 | 65 | 34.7 | 82 | 5.9 | 14 |  |
| Not at all | | Not very much | | Quite a lot | | Very much indeed | | 11. I feel restless as I have to be on the move: |
| Percent | No | Percent | No | percent | no | percent | no |  |
| 16.5 | 39 | 40.7 | 96 | 32.6 | 77 | 10.2 | 24 |  |
| Hardly at all | | Definitely less than I used to | | Rather less than I used to | | As much as I ever did | | 12. I look forward with enjoyment to things: |
| Percent | No | Percent | No | percent | no | percent | no |  |
| 3.00 | 7 | 19.9 | 47 | 50.00 | 118 | 27.1 | 64 |  |
| Not at all | | Not very often | | Quite often | | Very often indeed | | 13. I get sudden feelings of panic: |
| Percent | No | Percent | No | percent | no | percent | no |  |
| 15.3 | 36 | 34.3 | 81 | 36.4 | 86 | 14 | 33 |  |
| Very seldom | | Not often | | Sometimes | | Often | | 14. I can enjoy a good book or radio or TV program: |
| Percent | No | Percent | No | percent | no | percent | no |  |
| 8.1 | 19 | 18.6 | 44 | 29.2 | 69 | 44.1 | 1o4 |  |
| depression ranges over (0- 21); anxiety ranges over (0- 21); Higher scores indicate a greater level of anxiety or depression.  Anxiety score is a sum of responses for each of odd items (1, 3, 5, 7, 9, 11, and 13).  depression scale is a sum of responses for each of even items (2, 4, 6, 8, 10, 12, and 14), | | | | | | | | |
